# The effect of obesity on optic nerve sheath diameter in patients undergoing laparoscopic gynecological surgery: a prospective observational study

**DOI:** 10.1007/s00540-025-03482-1

**Published:** 2025-03-20

**Authors:** Hyerim Kim, Taikyung Seol, Jee-Eun Chang, Dongwook Won, Jung-Man Lee, Tae Kyong Kim, Eun Bi Park, Jin-Young Hwang

**Affiliations:** 1 Department of Anesthesiology and Pain Medicine, College of Medicine, SMG-SNU Boramae Medical Center, Seoul National University, Boramae-Ro 5, Dongjak-gu, Seoul, 156-707 Republic of Korea; 2 Department of Anesthesiology and Pain Medicine, Sheikh Khalifa Specialty Hospital, RAK, United Arab Emirates; 3 Department of Anesthesiology and Pain Medicine, Seoul National University Hospital, Seoul, Republic of Korea

**Keywords:** Obesity, Optic nerve sheath diameter, Pneumoperitoneum, Trendelenburg position

## Abstract

**Purpose:**

Pneumoperitoneum and the steep Trendelenburg position during laparoscopic gynecological surgery may increase intracranial pressure, which can be estimated using ultrasound measurement of the optic nerve sheath diameter (ONSD). In this study, we evaluated the effect of obesity on ONSD in patients undergoing laparoscopic gynecological surgery.

**Methods:**

Sixty-eight patients who underwent laparoscopic gynecological surgery were allocated to either the non-obese (*n* = 34) or obese (*n* = 34) groups. ONSD was assessed using ultrasound after anesthesia induction, at 30 and 60 min after pneumoperitoneum and Trendelenburg positioning, and at 10 and 60 min, and 24 h after desufflation and return to the supine position. Postoperative nausea and vomiting (PONV) and headache were evaluated 1 and 24 h after surgery.

**Results:**

ONSD increased significantly during pneumoperitoneum and Trendelenburg positioning in both groups (*P* < 0.001, respectively) and was higher in the obese group at each time point throughout and after surgery (*P* < 0.007, respectively). The increased ONSD during surgery returned to baseline 24 h after desufflation in the non-obese group, but not in the obese group. The incidence of PONV 1 h after surgery was significantly higher in the obese group than in the non-obese group (59% vs. 21%, respectively; *P* = 0.001). The incidence of PONV 24 h after surgery and postoperative headaches were not different between the two groups.

**Conclusion:**

ONSD was significantly higher in the obese group than in the non-obese group throughout and after laparoscopic gynecological surgery. The increased ONSD during surgery did not return to baseline even 24 h after desufflation in the obese group.

## Introduction

Laparoscopy is widely used in gynecological surgery due to its advantages, such as less blood loss, reduced postoperative pain, and faster recovery. However, laparoscopic gynecological surgery requires a steep Trendelenburg position and a CO_2_ pneumoperitoneum to secure the operator’s visual field. Pneumoperitoneum increases thoracoabdominal pressure, which decreases venous drainage from the brain and increases cerebral blood flow and intracranial pressure (ICP) [1]. During the steep Trendelenburg position, intrathoracic pressure also increases [2], and is transmitted to the intracranial space, thereby increasing ICP [3].

The optic nerve sheath diameter (ONSD) measurement via ultrasound is a non-invasive and reliable method for assessing ICP. There is a significant increase in ONSD during laparoscopic surgery [4–8]. Although an increase in ICP during laparoscopy rarely causes serious neurological complications, it has potential to induce postoperative nausea and vomiting (PONV) or headaches [9]. A study involving patients undergoing laparoscopic hysterectomy showed that patients with more extensive increases in ONSD had a higher incidence of PONV or postoperative headaches [6].

Obesity rates have risen significantly in the past few decades [10], and the number of obese patients undergoing laparoscopic gynecological surgery is increasing. An increase in ICP during laparoscopic surgery may be exacerbated by obesity. Obese patients tend to have higher abdominal pressure, which may further elevate ICP during the steep Trendelenburg position and pneumoperitoneum [1]. Obese patients present with greater increases in ONSD during laparoscopic surgery performed in the supine position than those of non-obese patients [11]. However, it remains unclear how obesity affects ONSD during laparoscopic surgery involving pneumoperitoneum and the steep Trendelenburg position. It is also unclear when the increased ONSD during surgery returns to baseline levels. In the present study, we investigated the effect of obesity on ONSD during and after laparoscopic gynecological surgery.

## Methods

This study was approved by the Institutional Review Board of our hospital (no. 20–2020-40). After approval of the study protocol and registration prior to patient enrollment at ClinicalTrials.gov (NCT04427085). After obtaining written informed consent, adults aged ≥ 19 years who were scheduled for laparoscopic gynecological surgery with an expected time of pneumoperitoneum and Trendelenburg positioning ≥ 1 h were recruited for this study. Exclusion criteria included preexisting neurological or cerebrovascular disease, history of ophthalmological or cerebrovascular surgery, or a history of brain tumor surgery. Patients were allocated to the non-obese group or the obese group based on whether their body mass index was ≥ 30 kg m^−2^ [12].

No premedications were administered. Standard monitoring included electrocardiography, pulse oximetry, non-invasive arterial pressure measurement, and bispectral index score. Anesthesia started with intravenous remifentanil (0.5 μg kg^−1^ min^−1^) and propofol (1.5 mg kg^−1^). Tracheal intubation was performed following achieving neuromuscular blockade with rocuronium (0.8 mg kg^−1^). Anesthesia was maintained using sevoflurane and a continuous infusion of intravenous remifentanil, targeting a bispectral index score value between 40 and 60. An arterial line was placed in the radial artery to monitor invasive arterial pressure. The mechanical ventilator was set to a tidal volume of 6–8 ml kg^−1^ of predictive body weight with a positive end-expiratory pressure (PEEP) of 5 cm H_2_O. Respiratory rate adjustments were made to maintain end-tidal carbon dioxide levels around 35 mmHg.

**Fig. 1:**
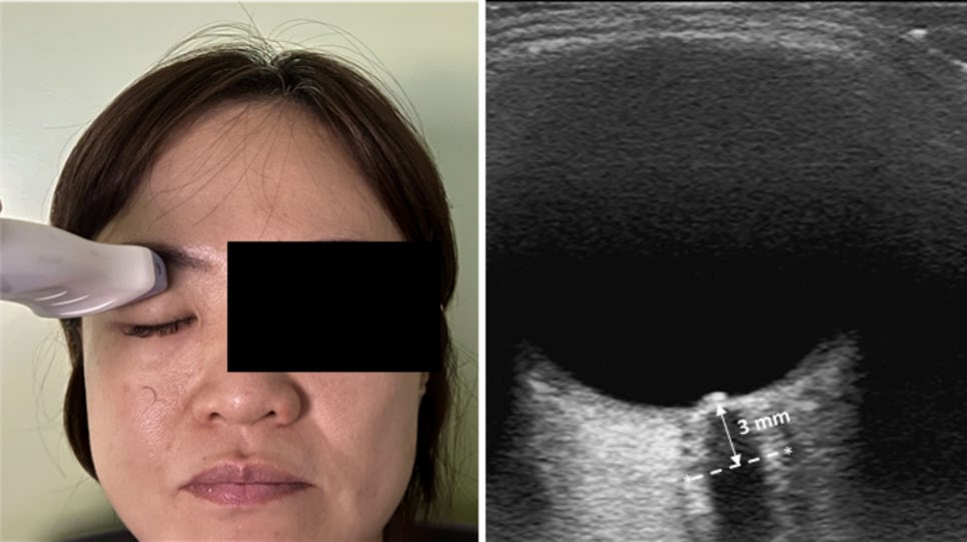
Ultrasound measurement of the optic nerve sheath diameter (ONSD). The probe was applied in the transverse plane on the eyelid, and the optic nerve was observed longitudinally behind the orbit at its widest diameter. The ONSD was assessed as the distance between the external borders of the hyperechoic area surrounding the optic nerve, 3 mm behind the retina

Once the cardiovascular status was stabilized after anesthesia induction and before starting surgery, the optic nerve sheath was evaluated in the supine position using a linear L12-3 probe (3 − 12 MHz) on a CX50 ultrasound system (Philips Healthcare, Andover, MA, USA). A transparent film was applied over the closed eyes, and a sterile gel was applied to the covered upper eyelids. For safety reasons, the mechanical index was set to 0.23, and the linear probe was placed on the eyelids without applying any pressure. The probe was applied in the transverse plane of the eyelid, and the optic nerve was observed longitudinally behind the orbit at its widest diameter. ONSD was measured 3 mm behind the eye globe, as described in previous studies [13, 14]. To measure ONSD, the width between the external borders of the hyperechoic area surrounding the optic nerve was determined (Fig. 1). Ultrasonographic measurements of ONSD were performed by a single researcher who had previous experience measuring ONSD in more than 50 cases.

ONSD was assessed at seven time points: T_Induction_, baseline after induction of anesthesia before artificial pneumoperitoneum and in the supine position; T_Pneumo+Tren15m_, 15 min after artificial pneumoperitoneum with CO_2_ insufflation and Trendelenburg position; T_Pneumo+Tren30m_, 30 min after pneumoperitoneum and Trendelenburg position; T_Pneumo+Tren60m_, 60 min after pneumoperitoneum and Trendelenburg position; T_Desuff+Sup10m_, 10 min after deflation and return to the supine position; T_Desuff+Sup60m_, 60 min after desufflation and return to the supine position; and T_Desuff+Sup24h_, 24 h after deflation and return to the supine position. The patient was angled at 30 degrees in the head-down position during the Trendelenburg position. The insufflation pressure was maintained at 12 mmHg. At each time point, the right and left ONSDs were measured twice, and peak inspiratory pressure, mean arterial pressure, and end-tidal CO_2_ were documented. Ramosetron (0.5 mg) was intravenously administered before the end of surgery, and an intravenous patient-controlled analgesia device containing 80 mg of morphine in 100 mL of isotonic normal saline solution was connected to the patient. Residual neuromuscular block was reversed using glycopyrrolate and neostigmine. After confirming full recovery, the trachea was carefully extubated. At 1 and 24 h after surgery, patients were asked if they had nausea, vomiting, or headaches. Mean arterial pressure was also recorded. Postoperative pain was assessed daily using a 100-point numerical rating scale (0: no pain, 100: worst pain imaginable). The primary outcome was ONSD at T_Pneumo+Tren60m_. Secondary outcomes were ONSD at each time point, the occurrence of PONV, and headaches after surgery.

All statistical analyses were carried out using statistical software SPSS version 27 (IBM Corp., Armonk, NY, USA). Continuous variables were tested for normality using graphical methods, such as histograms and Q–Q plots, as well as the Shapiro–Wilk test. Data are expressed as means (standard deviations), or numbers (percentages). ONSD over time within each group and between the two groups was analyzed using repeated-measures analysis of variance. A *P*-value < 0.05 was considered statistically significant. If significant differences in ONSD within each group or an interaction between ONSD and the group were found in repeated-measures analysis of variance, we planned to perform a post hoc analysis. To determine differences in ONSD between T_Induction_ and other time points within each group, paired t-tests were conducted, and the *P*-value was considered significant at *P* < 0.008 (0.05/6) to adjust for multiple comparisons. For differences between the two groups at each time point, an independent t-test or Mann–Whitney U test was performed, and a *P*-value < 0.007 (0.05/7) was considered significant. Categorical variables were analyzed using chi-square or Fisher’s exact tests. The Mann–Whitney U test or independent t-test was used to evaluate the differences between the non-obese and obese groups in terms of peak inspiratory pressure, mean arterial pressure, and end-tidal CO_2_ at each time point. A *P*-value < 0.05 was considered statistically significant.

The sample size was calculated based on a preliminary study in 24 patients showing that the mean ONSD at T_Pneumo+Tren60m_ was 4.73 (0.48) mm in the non-obese group and 5.14 (0.62) mm in the obese group. With a type I error of 0.05 and a power of 80%, it was calculated that 32 patients would be required per group. Considering a potential dropout rate of 10%, 35 patients were included per group.

## Results

**Fig. 2:**
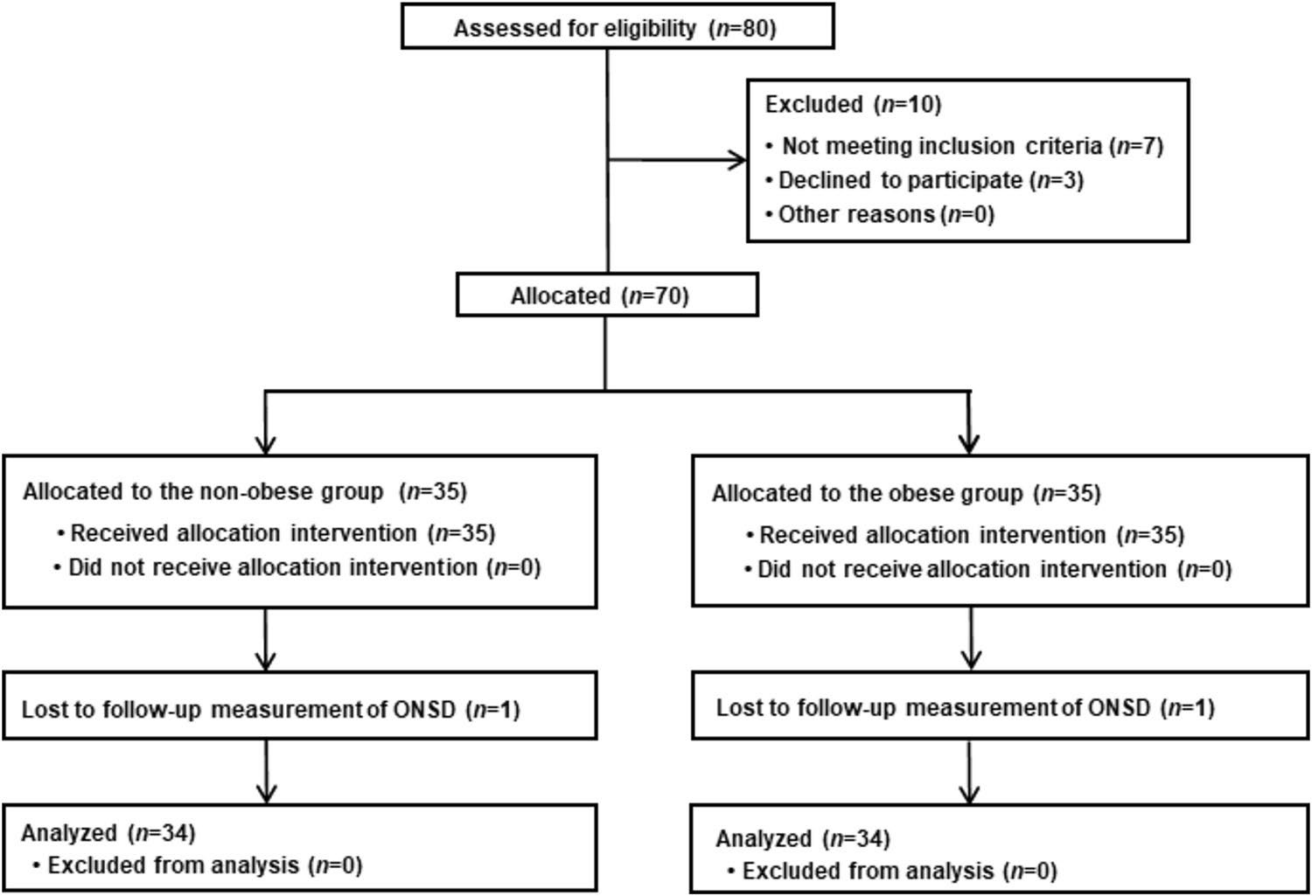
Study flow diagram. ONSD, optic nerve sheath diameter

Eighty patients were enrolled between August 2020 and May 2022. Of these, 10 patients were excluded because they either declined to participate or did not meet the inclusion criteria. Consequently, 70 patients were assigned to the two groups based on whether their body mass index was ≥ 30 kg m^−2^, including 35 patients in each group. Two patients were excluded due to missing data; therefore, 68 patients were ultimately included in the final analysis (Fig. 2). Table 1 shows patient- and surgery-related data are presented.

**Table 1:** Patient characteristics and data related to surgery

	Non-obese (*n* = 34)	Obese (*n* = 34)	*P*-value
Age (years)	41 (11)	39 (9)	0.585
Height (m^2^)	160 (6)	159 (4)	0.332
Weight (kg)	60 (9)	90 (7)	< 0.001
Body mass index (kg m^−2^)	24 (3)	36 (2)	< 0.001
ASA physical status (1/2)	18/16	0/34	< 0.001
Infusion rate of remifentanil (μg/kg/min)	0.31 (0.01)	0.30 (0.01)	0.220
Requirement for vasopressors (yes/no)	14/20	17/17	0.465
NRS on the day of surgery	26.1 (13.7)	22.6 (12.0)	0.086
NRS 1 day after surgery	29.4 (7.6)	28.8 (3.2)	0.072
Requirement for rescue medication (yes/no)	10/24	8/26	0.582
Duration of surgery (min)	123 (28)	125 (32)	0.873
Duration of anesthesia (min)	144 (29)	147 (31)	0.853

Data are expressed as means (SD) or as the number of patients

*ASA* American society of anesthesiologists, *NRS* numerical rating scale (0: no pain, 100: worst pain imaginable)

**Fig. 3:**
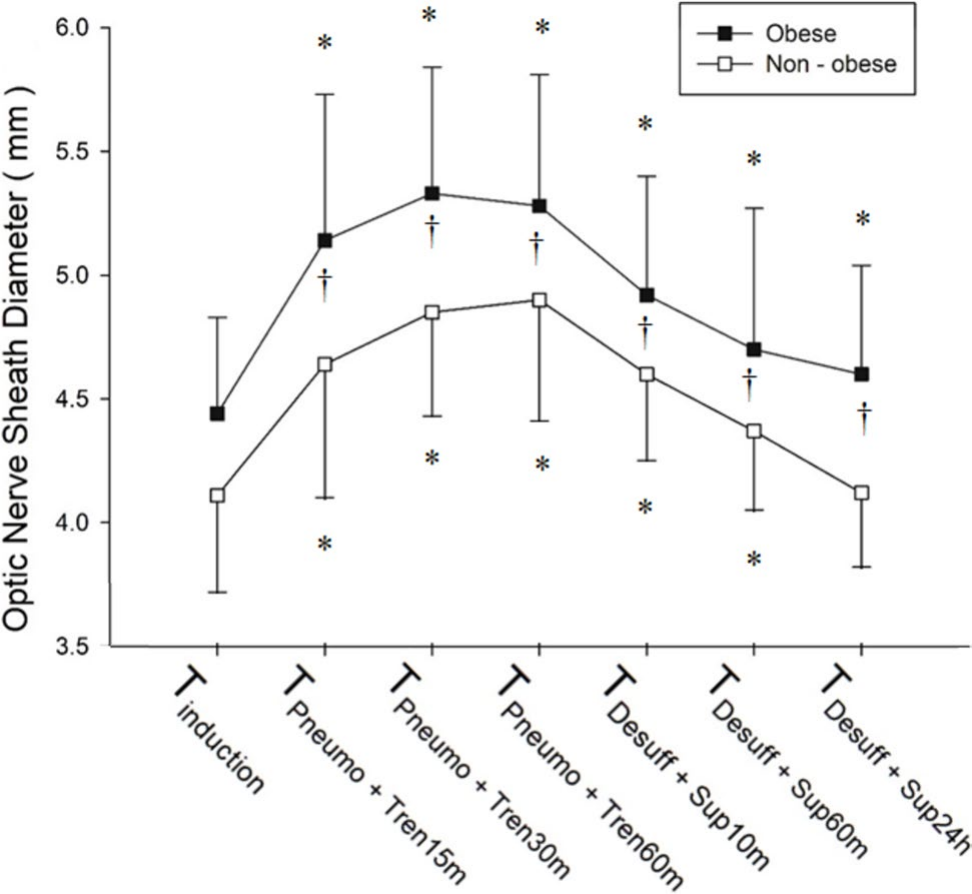
The change in optic nerve sheath diameter (ONSD) (mm) throughout and after surgery. Data are expressed as means. The error bars represent one SD. T_Induction_, baseline after induction of anesthesia before artificial pneumoperitoneum and in the supine position; T_Pneumo+Tren15m_, 15 min after artificial pneumoperitoneum with CO_2_ insufflation and Trendelenburg position; T_Pneumo+Tren30m_, 30 min after pneumoperitoneum and Trendelenburg position; T_Pneumo+Tren60m_, 60 min after pneumoperitoneum and Trendelenburg position; T_Desuff+Sup10m_, 10 min after deflation and return to the supine position; T_Desuff+Sup60m_, 60 min after desufflation and return to the supine position; T_Desuff+Sup24h_, 24 h after deflation and return to the supine position. *P < 0.008 vs. T_Induction_ in each group, †*P* < 0.007 vs. non-obese group

The ONSDs during the perioperative period are shown in Fig. 3. ONSD changed over time in both groups (*P* < 0.001, respectively). In the non-obese group, ONSD was significantly increased at T_Pneumo+Tren15m_, T_Pneumo+Tren30m_, T_Pneumo+Tren60m_, and T_Desuff+Sup10m_ (*P* < 0.001, respectively) and T_Desuff+Sup60m_ (*P* = 0.002) compared to that measured at T_Induction_, and returned to baseline at T_Desuff+Sup24h_ (*P* = 0.918). In the obese group, ONSD was also significantly increased during and after the release of Trendelenburg positioning and pneumoperitoneum (at T_Pneumo+Tren15m_, T_Pneumo+Tren30m_, T_Pneumo+Tren60m_, T_Desuff+Sup10m_, and T_Desuff+Sup60m_) compared to that at T_Induction_ (*P* < 0.001, respectively), but did not return to baseline even at T_Desuff+Sup24h_ (*P* < 0.001). There was a statistically significant interaction between ONSD and group (*P* = 0.012). The ONSD was significantly higher in the obese group than in the non-obese group at each time point [T_Induction_: 4.44 (0.39) mm vs. 4.11 (0.39) mm, respectively; *P* = 0.001, T_Pneumo+Tren15m_: 5.14 (0.59) mm vs. 4.64 (0.54) mm, respectively; *P* < 0.001, T_Pneumo+Tren30m_: 5.33 (0.51) mm vs. 4.85 (0.42) mm, respectively; *P* < 0.001, T_Pneumo+Tren60m_: 5.28 (0.53) mm vs. 4.90 (0.49) mm, respectively; *P* = 0.002, T_Desuff+Sup10m_: 4.92 (0.48) mm vs. 4.60 (0.35) mm, respectively; *P* = 0.003, T_Desuff+Sup60m_: 4.70 (0.57) mm vs. 4.37 (0.32) mm, respectively; *P* < 0.001, T_Desuff+Sup24h_: 4.60 (0.44) mm vs. 4.12 (0.30) mm, respectively; *P* < 0.001].

**Fig. 4:**
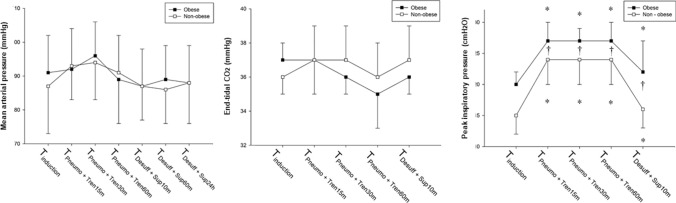
The change in mean arterial pressure, end-tidal CO_2_, and peak inspiratory pressure during perioperative period. Data are expressed as means. The error bars represent one SD. T_Induction_, baseline after induction of anesthesia before artificial pneumoperitoneum and in the supine position; T_Pneumo+Tren15m_, 15 min after artificial pneumoperitoneum with CO_2_ insufflation and Trendelenburg position; T_Pneumo+Tren30m_, 30 min after pneumoperitoneum and Trendelenburg position; T_Pneumo+Tren60m_, 60 min after pneumoperitoneum and Trendelenburg position; T_Desuff+Sup10m_, 10 min after deflation and return to the supine position; T_Desuff+Sup60m_, 60 min after desufflation and return to the supine position; T_Desuff+Sup24h_, 24 h after deflation and return to the supine position. *P < 0.05 vs. T_Induction_ in each group, †*P* < 0.05 vs. non-obese group

Peak inspiratory pressure, mean arterial pressure, and end-tidal CO_2_ during the perioperative period are shown in Fig. 4. No significant differences were observed in mean arterial pressure and end-tidal CO_2_ between the two groups at any time point (*P* > 0.05, respectively). However, the peak inspiratory pressure was significantly higher in the obese group than in the non-obese group at each time point (*P* < 0.001, respectively).

The incidences of PONV and headache after surgery are shown in Table 2. The incidence of PONV at 1 h after surgery was significantly higher in the obese group than in the non-obese group (59% vs. 21%., respectively; *P* = 0.001). The incidence of PONV at 24 h after surgery was not different between the obese and non-obese groups (12% vs. 9%, respectively; *P* = 0.690). There was no significant difference in the incidence of headaches between the two groups at 1 and 24 h after surgery (*P* > 0.05, respectively).

**Table 2:** The incidences of postoperative nausea and vomiting (PONV) and headache

	Non-obese (*n* = 34)	Obese (*n* = 34)	*P-*value
PONV at 1 h	7 (21)	20 (59)	0.001
PONV at 24 h	3 (9)	4 (12)	0.690
Headache at 1 h	0 (0)	0 (0)	-
Headache at 24 h	6 (18)	3 (9)	0.283

Data are expressed as the number of patients (%)

## Discussion

This study showed that ONSD was significantly increased during laparoscopic gynecological surgery in both obese and non-obese groups and was significantly higher in the obese group than in the non-obese group when measured after the induction of anesthesia, during and after the release of artificial pneumoperitoneum and the Trendelenburg position. Furthermore, the increased ONSD during laparoscopic gynecological surgery returned to baseline 24 h after desufflation and the return to the supine position in the non-obese group, even so did not return to baseline even 24 h after desufflation in the obese group. The incidence of PONV 1 h after surgery was notable higher in the obese group than in the non-obese group. However, the occurrence of PONV 24 h after surgery and postoperative headaches did not differ based on obesity.

Our findings of a significant increase in ONSD during laparoscopic gynecological surgery are consistent with those of previous studies [4, 6, 15]. These studies showed an increase in ONSD during pneumoperitoneum for laparoscopic gynecological surgery, mostly in non-obese patients. In the present study, we compared ONSD in both obese and non-obese patients during surgery and up to 24 h after desufflation and found that ONSD was significantly higher in obese patients than in non-obese patients throughout and after surgery. ONSD was significantly greater in the obese group than in the non-obese group from the baseline because obese patients tend to have higher thoracoabdominal pressure, which may affect ICP, thereby being reflected in ONSD from the baseline, although it is within the normal range. Our finding that obese patients had higher ONSD after the induction of anesthesia and during pneumoperitoneum than non-obese patients is consistent with the results of a study comparing the change in ONSD in obese and non-obese patients who in supine position underwent laparoscopic procedures [11]. In that study, the ONSD was assessed prior to pneumoperitoneum, during insufflation at 15 and 30 min, and immediately after deflation of pneumoperitoneum. The ONSD was significantly higher in obese patients at each time point; however, the mean body mass index of all patients included in that study was described as 30 kg m^−2^, but the criteria for obesity and the body mass index of each group were not clearly described. Furthermore, in that study, the Trendelenburg position was not applied during laparoscopic surgery, and the ONSD increased over time and returned to baseline levels following abdominal deflation. In our study, the increased ONSD during surgery returned to baseline 24 h after surgery in the non-obese group, but not in the obese group, which is consistent with a previous study showing that the ONSD remained elevated compared to the baseline value despite CO_2_ desufflation when measured 10 min after the release of pneumoperitoneum [6]. Most other studies observed the ONSD before, during, and after pneumoperitoneum during surgery, and did not conduct long-term follow-up [4–7, 11, 15]. To the best of our knowledge, this is the first study to observe the change in ONSD not only during surgery but also up to 24 h after desufflation.

Another important issue in interpreting our results is that the absolute ONSD value of obese patients was already elevated compared to those of non-obese patients at T_induction_. Furthermore, the observed increases in ONSD following their implementation seem to be similar between obese and non-obese patients. Therefore, some specific factors might have contributed to the ONSD expansion in obese patients during the T_induction_ stage, although we did not evaluate them. Furthermore, ONSD values in obese patients had not returned to baseline even by T_Desuff+Sup24h_. It also remains uncertain whether this outcome is attributable to the effects of pneumoperitoneum and the Trendelenburg position or to other factors already present during induction of anesthesia.

In the present study, the incidence of PONV was significantly higher in obese patients 1 h after surgery but did not differ between the two groups 24 h after surgery [16, 17]. The higher incidence of PONV 1 h after surgery might be due to increased ICP, as indicated by the higher ONSD following the steep Trendelenburg position and pneumoperitoneum in the obese group. It has been reported that the extent of the increase in ONSD during the procedure is significantly correlated with PONV and headaches occurring within the first 3 h of recovery [6]. However, our study was not powered to detect the relationship of ONSD with PONV; therefore, further studies are needed to validate this hypothesis.

The ONSD is thought to be correlated with ICP levels, and the reported cutoff for detecting elevated ICP ranges from 4.8 to 5.6 mm [13, 18, 19]. Mean baseline ONSD after the induction of anesthesia was 4.11 mm in the non-obese group and 4.44 mm in the obese group. However, during the Trendelenburg position and pneumoperitoneum, the ONSD significantly increased, with the mean peak ONSD reaching 5.33 mm at T_Pneumo+Tren30m_ in the obese group and 4.90 mm at T_Pneumo+Tren60m_ in the non-obese group. In the present study, the obese group had a higher ONSD than the non-obese group at each time point from the baseline. Although the change in ONSD from the baseline to each time point may not be different between the two groups, we focused on the absolute value of ONSD at each time point between the two groups because the absolute value of ONSD rather than the degree of increase in ONSD is more meaningful to assess increased ICP. The obese group had a significantly higher ONSD, which may indicate an increased ICP. Therefore, although pneumoperitoneum and Trendelenburg positioning are required to secure the surgical field during laparoscopic gynecological surgery, excessive insufflation or a steep Trendelenburg position should be avoided because of the possibility of increased ICP, particularly in obese patients. Furthermore, low arterial pressure should be avoided to ensure adequate cerebral perfusion. Higher airway pressure may also affect ONSD although we did not evaluate it in this study. Therefore, it is important to suppress an increase in airway pressure for ICP management in obese patients, and further studies on the relationship between airway pressure affected by obesity and respiratory management, and the change of ONSD are required.

Additionally, a difference of less than 1 mm in ONSD between the two groups might be considered insignificant. However, the standard for increased ICP is divided in mm, and even a slight difference in ONSD may reflect increased ICP; therefore, our results cannot be ignored.

In the present study, a PEEP of 5 cmH_2_O was applied to both groups because PEEP increases intrathoracic pressure. However, obese patients may need higher PEEP to avoid lung collapse, although an adequate level of PEEP for obese patients has not been clearly determined [20, 21]. Therefore, if higher PEEP is applied to obese patients during gynecological laparoscopic surgery, the ONSD could be much higher than in non-obese patients.

This study had several limitations. First, the operator who measured ONSD was not blinded to the time points of ONSD assessment. Furthermore, the operator evaluated the ONSD without knowing the patient’s BMI for blindness, but might notice the obesity status of the patients although he or she could not accurately determine obesity levels. However, the operator tried to measure ONSD according to a standardized study protocol. Second, a single operator assessed the ONSD, which makes the interpretation of the results challenging. However, it would minimize inter-observer variability. Third, the mean body mass index of obese group in this study was 36 kg m^−2^, and the results may differ between obese and morbidly obese patients (body mass index ≥ 40 kg m^−2^). Fourth, this study was conducted on an Asian population. Therefore, our results may not be generalizable to other ethnic groups. Fifth, the baseline ONSD was assessed after the induction of anesthesia, according to previous studies [6, 8, 11, 15], but not in the awake state before the induction of anesthesia, and the recovery of ONSD after surgery was evaluated based on baseline ONSD. Anesthesia may affect ICP. Therefore, if the baseline ONSD was set before the induction of anesthesia, the time point of return to baseline ONSD after surgery might differ from the current results. Finally, the mean operation time, including pneumoperitoneum and Trendelenburg positioning, was approximately 120 min in both groups. According to a previous study [22], prolonged intracranial hypertension affects ONSD reversibility. Therefore, if the duration of pneumoperitoneum and Trendelenburg positioning were longer or shorter, the timing of recovery to baseline ONSD might vary.

## Conclusions

ONSD significantly increased during laparoscopic gynecological surgery in both obese and non-obese groups, and ONSD was significantly higher in the obese group than in the non-obese group throughout and after surgery. Furthermore, the increased ONSD during pneumoperitoneum and the Trendelenburg position did not return to baseline, even 24 h after desufflation and the return to the supine position in the obese group. The higher incidence of PONV at 1 h after surgery in the obese group may be due to a higher ONSD. Therefore, excessive and prolonged pneumoperitoneum or a steep Trendelenburg position should be avoided to prevent considerable increase in ICP, particularly in obese patients undergoing laparoscopic gynecological surgery.
